# Historical Review of the Medical Research Committee

**Published:** 1919

**Authors:** Kenneth Taylor

**Affiliations:** Honorary Secretary


### HISTORICAL REVIEW OF THE MEDICAL RESEARCH COMMITTEE

### Of the American Red Cross in France.

Compiled from the Minutes of the Secretary and the Reports in *War Medicine.*

It is fitting that in this final mumber of *War Medicine* a review of the research activities of the Red Cross should be published. The Red Cross has generously contributed to aid in the solution of medical and surgical problems which have arisen during the war, and has taken an active part in the distribution to the Army Medical Services of such medical knowledge as was available.

Its main activities have been promoted and directed under the guidance of its medical adviser, Colonel Alexander Lambert, and it is largely due to his untiring energy and scientific insight that such success has been attained.

The work has been chiefly done through the medium of a committee of inter-allied composition, the Research Committee of the A. R. C. It is with the formation and growth of this and its dependent organizations that the present history is concerned.

Before America entered into the war, it was recognized by many of the medical men working in military hospitals that a greater amount of co-operation between the professional services of the allied armies would lead to a more rapid advance of medical knowledge, and consequently to a more efficient treatment of the war casualties. The formation of the Inter-allied Surgical Conference was perhaps the first manifestation of this sentiment. This, however, was limited to the formal consideration of surgical problems, and the meetings were conducted in French, which unfortunately limited its usefulness for many Americans.

With the arrival of the American Red Cross Commission in France, there seemed to be an opportunity to promote under its management the needed research and educational program. In August 1917, after discussing this matter with Colonel Joseph A. Blake, who was at that time in charge of what was to become the American Red Cross Military Hospital N° 2, Lt.-Colonel Kenneth Taylor proposed to Colonel Lambert, then Chief Surgeon of the American Red Cross, the establishment of the following organization : a Committee consisting of members of the Red Cross Commission and of the American Army Medical Corps, to be appointed by the Red Cross for the purpose of stimulating research, and aiding in the distribution of the medical information acquired by America's allies during the first years of the war. This plan involved :

First : The establishment of a research laboratory to serve as a centre for Red Cross or Army medical officers who desired an especially well equipped laboratory for research work, and to aid in the preservation of material of scientific educational value.

Second : The establishement of a médical library consisting of current periodicals and reference books for the use of medical men in Paris.

Third : The publication of reports of medical articles appearing in various European journals, to distribute in this manner a condensed review of the medical activities in the various armies.

Fourth : The institution of monthly meetings of Army medical officers for the purpose of discussing medical problems of the war, and hearing reports from the medical officers of the French and British Armies.

The project was approved by Colonel Lambert and the Committee was shortly afterwards appointed by Colonel Grayson Murphy, Commissioner of the Red Cross in Europe, to develop these activities, and to advise on the method of utilizing the funds set apart for this educational and medical research program.

This Committee was composed as follows :

Colonel JOSEPH A. BLAKE.
Lt.-Col. WALTER CANNON, Chairman.
Colonel GEORGE W. CRILE.
Colonel HARVEY CUSHING.
Major-General M. W. IRELAND.
Colonel ALEXANDER LAMBERT.
Doctor JAMES A. MILLER, A. R. C.
Colonel J. F. SILER.
Colonel R. P. STRONG.
Lt.-Col. HOMER SWIFT.
Lt.-Col. KENNETH TAYLOR, Secretary.
Doctor W. C. WHITE, A. R. C.

This was the nucleus for the American Red Cross Research Committee, which later developed into an informal council of the medical men of the Allied Armies. Its appointment was announced at a meeting of the Medical Advisory Committee of the American Red Cross. The new Committee met for the first time on the 25th of October, 1917.

It may be interesting to follow briefly the development of the several projects of this Committee.

THE RESEARCH LABORATORY,

The American Red Cross Research Laboratory N° 1 was established at once under the direction of Lt.-Col. Taylor at the Red Cross Military Hospital, 6 rue Piccini, Paris, in co-operation with the Robert Walton Goelet Research Laboratory which had been in operation since early in the war. This laboratory continued to function until the hospital was closed in January, 1919. Besides conducting a considerable a mount of practical research work, it served during that time various purposes. It was used for the training of laboratory workers who were transferred to other Red Cross or Military Hospitals. It conducted the greater part of the routine laboratory work for the Red Cross hospital activities in the Paris district. It served for nearly a year as the United States Army base laboratory for the district of Paris, when it was the centre for the distribution of supplies and the practice of technical laboratory examinations for a large number of army organizations. In the spring of 1918 Major H. E. Robertson of the Museum Section of the Army Medical Corps made the laboratory his headquarters in Paris, and through his activities the project of collecting pathological material of educational value was realized. The laboratory also served as the office for the secretary of the Committee, and as the editorial office of the A. R. C.'s chief medical publication (*War Medicine*) during the first few months after its appearance.

### The Medical Library.

At the time of the organization of the laboratory, the Red Cross Medical Library was established in the same quarters. This library contained the leading French, English and American medical periodicals, and proved to be of great value to medical officers in the Paris district. It was also used as the distributing centre for thousands of reprints and official reports issued by the British Medical Research Committee. These valuable publications were regularly and systematically forwarded to American Army organizations. The library was maintained until the hospital closed in January 1919, when it was transferred to 12 Place Vendome and incorporated in the central reference library of the Red Cross.

### Publications.

The work of getting out a monthly review of medical literature was started at once. A small committee consisting of Colonel Lambert, Colonel R. P. Strong, and Lt.-Colonel Taylor was appointed to direct it. With the editorial assistance of Dr. E. B. Watson, the journals and publications received at the library were reviewed, abstracted and published in the form of the *Medical Bulletin*. Dr. Watson had a large share in this work, and the success of the journal from first to last has been considerably enhanced by his skill and interest in the project. This journal was distributed from the outset not only to the American Expeditionary Forces, but also to the British, French and Italian Armies, and to the allied medical men in more distant countries. This bulletin started with a modest circulation of 2000 copies. The demand, however, grew so rapidly that it was soon increased in size, and the circulation extended. In a few months the publication of the paper had become such a formidable undertaking that the original staff proved inadequate, and the journal was turned over to the direction of Colonel Seale Harris, as Editor; the staff was much increased, and new offices acquired. The name *Medical Bulletin* was changed to *War Medicine*, and the journal still further increased in size. The number of copies published and distributed each month was raised to 8000. In Colonel Harris' hands it quickly grew into an efficient means of condensing and distributing promptly valuable medical information throughout the Armies.

### Medical Meetings.

One of the most valuable activities of the Research Committee was the institution and direction of the monthly meetings of the medical men of the allied forces. With the co-operation of General Ireland, Chief Surgeon of the American Expeditionary Forces, orders were given representatives from the various army medical formations to attend these meetings two days each month, and to take part in the discussions of various important problems with which the Army was confronted.

The first meetings were held at the American Red Cross Military Hospital N° 2, 6, rue Piccini, but the attendance grew so large that after the first three gatherings it was found necessary to take larger quarters, and they were transferred first to the Pasteur Institute, and later to rooms at the Continental Hotel.

These meetings proved to be of inestimable educational value. Many critical problems were discussed and the accumulated experience of the leading medical officers of our Allies was brought to the attention of our own Medical Corps in a direct and practical manner.

The first meeting held on December 11-12, 1917, at 6 rue Piccini, was really an event in the medical history of the war. There were present about 150 American medical officers, and visitors from the British and French forces. Colonel Lambert, as Chief Surgeon of the Red Cross, opened the session and introduced Colonel Murphy, Commissioner for Europe, who wished success to the entreprise and welcomed the allied medical officers who had come to address the assembly. He predicted that these meetings would develop an invaluable spirit of co-operation among the medical corps of the allied armies, and that this co-operation would be of especial value to our own medical men who were comparatively inexperienced in the treatment of war casualties.

Colonel Blake took the chair throughout the meeting. Two great problems, Gas Gangrene and Trench Fever, which were new to the Americans, were presented by leading British and French authorities on those subjects.

The subject of Gas Grangrene was introduced by General Cuthbert Wallace of the British Forces. He was followed by Dr. Sacquépée, who discussed the French experience with the disease. Captain Henry and Major McNee, both of the British Medical Corps, then discussed the pathology and bacteriology of the condition. These speakers were followed by an open discussion in which Dr. Weinberg and Lt.-Colonel Taylor took part.

The subject of Trench Fever was admirably presented by Major McNee, and discussed by Colonel Strong, Lt.-Col. Swift, and Lt.-Col. Roger Lee of the American Forces, and by General Thompson and Major McNee of the British Army.

This was the first and one of the most successful of a series of meetings. Like those which followed, it was divided into three sessions. Eleven meetings were held, and a wide range of subjects were discussed by the most competent men in the Armies.

As most of the papers presented at the meetings have been published in the *Medical Bulletin* or *War Medicine*, a list of only the chief subjects need be given here. The following gives an idea of the ground covered :

| | | |
|---|---|---|
| First Session. | December 1917. | Gas Gangrene. |
| | | Trench Fever. |
| Second Session. | January 1918. | Tetanus. |
| | | Scabies and Pediculosis. |
| | | Louse Problem. |
| Third Session. | February 1918. | Cerebro-spinal Meningitis. |
| Fourth Session. | March 1918. | Traumatic Shook. |
| | | Hemorrhage and Transfusion. |
| | | Anesthesia. |

| | | |
|---|---|---|
| Fifth Session. | April 1918. | Dysentery (Bacillary and Amebic).<br>Report of Trench Fever Commission.<br>Venereal Prophylaxis. |
| Sixth Session. | May 1918. | Military Orthopedics.<br>Aviation. |
| Seventh Session. | June 1918. | Wounds of the Chest.<br>War Psychoneuroses.<br>Gas Poisoning. |
| Eighth Session.] | July 1918. | Treatment of Fractures of the Femur.<br>Sources of Wastage of Men in War.<br>Transportation of Wounded Men. |
| Ninth Session. | September 1918. | Conference on Surgery in Battle Areas.<br>Acute Respiratory Infections.<br>Field Sanitation. |
| Tenth Session. | October 1918. | Trench Foot.<br>Tetanus.<br>Restoration of the Sick and Woundel to the Line.<br>Streptococcus Infections of the Respiratory Tract. |
| Eleventh Session. | November 1918. | Conference on Tuberculosis of the Lungs.<br>Conference on Problems Relating to the Area of Advance. |

### The Committee.

No discussion of the activities of the Research Committee would be complete without a mention of the sympathy, co-operation and practical aid given by General Ireland, then Chief Surgeon of the American Expeditionary Forces. It was only through his assistance that the Committee was able to organize and successfully carry through its work.

Perhaps no more interesting group of men ever gathered together to discuss medical problems than that which attended the meetings of the Committee. It was this Committee which decided upon programs for the Society meetings, discussed the experiences presented at these meetings, and determined what lines of work were most likely to be profitable.

At the first meeting on October 25th, 1917, only part of the small nucleus of the Research Committee was present. At that time Colonel Lambert was elected vice-chairman of the Committee, and Lt.-Colonel Taylor, secretary. [Lt.-Colonel Cannon, had been named chairman when the Committee was appointed.] The policy of establishing special research laboratories to attack single, definitely outlined problems was then decided upon, and several problems were discussed. At this first meeting the need of research in Trench Fever was pointed out by Col. Strong and he was asked for a report and recommendations as to how to proceed. The secretary's proposal to hold monthly meetings of the Society was approved, and authorization given him to put them into operation and arrange the programs.

Twenty-three meetings were held in all. After the first four sessions Lt.-Colonel Cannon took up his work as chairman of the Committee and continued to act in that capacity until the last meeting. Lt.-Colonel Taylor continued the work of secretary of the Committee and the directorship of the *Medical Bulletin* for the first nine months, when he was transferred from the position of active, to honorary secretary, and turned over to Lt.-Col. Seale Harris, who had been brought over to France for this work, both the Committee work and the supervision of the Bulletin. Lt.-Col. Harris was formally appointed editor of the journal and secretary of the Committee, and continued to act in these capacities until the Committee was dissolved.

The problems discussed in the meetings of the Committee are fairly well illustrated by the programs of Society meetings, but aside from these topics many other important administrative difficulties and scientific problems were discussed and frequently solved by this Committee.

The Committee offered exactly what was needed in the medical organization of the Allies—an informal meeting place where the leaders of the medical services of the various armies could talk over their troubles. Many pleasant friendships grew out of this association, and a co-operative spirit was developed which made the solution of difficult situations easily obtainable. Some of the critical problems of the war could not be readily handled within one army organization, and this committee turned into an allied council chamber on many occasions.

Special sub-committees, in which were represented the French, British and American services, were frequently established to investigate important problems.

The first of these special committees was that appointed to study trench fever. This committee under the leadership of Colonel Strong, and with the co-operation of the British, completed one of the most clean-cut and valuable investigations in the medical history of the war, namely, the method of transmission and the nature of the virus in this disease.

A complete report of the interesting work of this committee has been published in book form, and has been distributed by the Research Committee.

A sub-committee on gas gangrene under the leadership of General Cuthbert Wallace, B. E. F., also made good progress. It was this committee which, by bringing together American and British work on gas gangrene, cleared up many misconceptions as to the pathology of this disease. It conducted, under General Wallace's supervision, an exhaustive trial of the usefulness of antitoxins in this formidable disease, and directed the production of sera along more practical lines than those previously followed.

A committee on shock was appointed, with Lt.-Colonel Walter Cannon as chairman, and under his direction carried out valuable researches in the treatment of traumatic shock.

A committee of inter-allied composition met and discussed the standardization of the treatment for fractures of the femur.

A committee was appointed to determine as far as possible the best treatment for infected wounds before secondary suture, and some interesting studies were reported to the general committee.

A dysentery committee was appointed, which met and discussed the control of that disease in the armies.

Several other committees were formed which, either because of difficulties in meeting or on account of the cessation of hostilities, made no formal reports, but they brought much valuable information, about the subjects which they were appointed to study, to the Research Committee for discussion.

The Committee itself grew rapidly in size. After the first few meetings of the original group, new members were added at almost every session, until at the time of the last meeting in November, 1918, the following was the composition of the Committee. The list represents only a small part of those who met with the Committee, and gave generously of their experience and advice.

Prof. GUISEPPE BASTIANELLI, Italian Army.
Col. JOSEPH A. BLAKE, Am. E. F.
Gen. Sir JOHN ROSE BRADFORD, B. E. F.
Maj. P. K. BROWN, A. R. C.
Lt.-Col. WALTER CANNON, Am. E. F.
Prof. A. CASTELLANI, Italian Army.
Col. G. W. CRILE, Am. E. F.
Col. S. L. CUMMINS, B. E. F.
Col. HARVEY CUSHING, Am. E. F.
Col. T. R. ELLIOTT, B. E. F.
Lt.-Col. HAVEN EMERSON, Am. E. F.
Gen. J. M. T. FINNEY, Am. E. F.
Sir WALTER FLETCHER, Secretary of British Medical Research Committee.
Lt.-Col. SEALE HARRIS, Secretary.
Maj.-Gen. M. W. IRELAND, Am. E. F.
Col. ALEXANDER LAMBERT, Vice Chairman.
Prof. C. LEGROUX, French Army.
Col. Sir WILLIAM LEICHMAN, B. E. F.
Col. W. D. McCAW, Am. E. F.
Dr. JAMES A. MILLER, A. R. C.
Maj. EDOUARD RIST, French Army.
Maj. H. A. SHAW, Am. E. F.
Col. J. F. SILER, Am. E. F.
Colonel R. P. STRONG, Am. E. F., Chairman.
Lt.-Col. HOMER SWIFT, Am. E. F.
Lt.-Col KENNETH TAYLOR, Am. E. F., Hon. Secretary.
Gen. W. S. THAYER, Am. E. F.
Gen. CUTHBERT WALLACE, B. E. F.
Dr. W. C. WHITE, A. R. C.

The Committee hopes that it has dissolved only in name, and that the friendships and associations which have been so pleasant a feature of these meetings during the past 18 months will continue in the future, and that they may form the basis for a closer union of the medical profession of France, England, and America.

KENNETH TAYLOR, Lt.-Col., M. C.
Honorary Secretary.

